# Whole genome sequences of novel *Staphylococcus epidermidis*, *Streptococcus salivarius*, and *Streptococcus anginosus* strains isolated from human gastric biopsy tissue

**DOI:** 10.1128/mra.01136-25

**Published:** 2026-02-19

**Authors:** Anthony Mannion, Zeli Shen, JoAnn Dzink-Fox, M. Blanca Piazuelo, Keith T. Wilson, Richard M. Peek, James G. Fox

**Affiliations:** 1 Division of Comparative Medicine, Massachusetts Institute of Technology 2167, Cambridge, Massachusetts, USA; 2 Vanderbilt University Medical Center 12328, Nashville, Tennessee, USA; Nanchang University, Nanchang, Jiangxi, China

**Keywords:** gastric microbiome, gastric disease, gastric cancer, non-*Helicobacter pylori*, *Staphylococcus*, *Streptococcus*

## Abstract

Three novel bacterial strains, *Staphylococcus epidermidis*, *Streptococcus salivarius*, and *Streptococcus anginosus*, were cultured from human gastric biopsy tissue from Colombian, South America patients with a high risk for gastric cancer. Bacteria were sequenced using Illumina MiSeq technology for phylogenetic characterization and identification of genetic mechanisms conferring pathogenic or protective potential.

## ANNOUNCEMENT

While *Helicobacter pylori* infection is critical for driving the progression of gastric disease and carcinogenesis, recent studies have indicated an important influence of the gastrointestinal microbiome. Previous studies in our laboratory investigating a cohort of high-risk gastric cancer patients in Colombia, South America, have identified changes in the gastric microbiota composition associated with promotion/protection of disease progression (1–4). Specifically, we have identified that *Staphylococcus epidermidis* and *Streptococcus salivarius* were frequently isolated from gastric biopsy specimens from patients located in Tumaco and Túquerres, Colombia (2). Subsequent *in vivo* studies using germfree INS-GAS mice found that *S. salivarius* co-infection with *H. pylori* induced significantly higher gastric pathology than in *H. pylori*-mono-infected mice, whereas *S. epidermidis* co-infection caused significantly lower *H. pylori*-induced pro-inflammatory cytokine responses than in *H. pylori*-mono-infected mice (2). Recently, Fu et al. (5) identified *Streptococcus anginosus* in gastric tissue and found that infection in mice promotes gastric inflammation, atrophy, and tumorigenesis. In our Colombian cohort, we have also cultured *S. anginosus* strains from gastric biopsy specimens and have shown that *S. anginosus* infection augments *H. pylori*-induced gastric disease in germfree INS-GAS mice (4). Herein, we describe the whole genome sequence characterization of these *S. epidermidis*, *S. salivarius*, and *S. anginosus* strains.

Endoscopic collection of antral gastric biopsy samples from human subjects was reviewed and approved by the Universidad del Valle (IRB # 024-09) and Vanderbilt University (IRB # 071000). Gastric biopsy samples were homogenized using tissue grinders in BHI media (BD, catalog no. 238400) with 20% glycerol. Homogenates were plated onto 5% sheep blood agar plates (Remel, catalog no. R01202) and incubated at 37°C in 5% CO_2_ for 24–48 h to grow colonies. Genomic DNA was isolated using the MasterPure Complete DNA and RNA Purification Kit (Epicenter) for *S. epidermidis* and *S. salivarius* or the High Pure PCR Product Purification Kit (Roche Laboratories) for *S. anginosus*. Genomic DNA was prepared for Illumina MiSeq sequencing (2 × 250 bp) using the Nextera Flex library prep kit. Default parameters were used for all bioinformatics tools. Raw sequenced reads were decontaminated of adapter sequences and quality trimmed using BBDuk (version: ≥37.17; parameters: ktrimr, k23, mink11, hdist1, tpe, tbo, qtrimrl, trimq10, and qin33) (6) followed by *de novo* contig assembly on the Bacterial and Viral Bioinformatics Resource Center (BV-BRC) (7) using SPAdes (version: 3.10.0) for *S. epidermidis* and *S. salivarius* or Unicycler (version: 0.4.8) for *S. anginosus*. Gene annotation was performed using RASTtk (2020-04-06) hosted by BV-BRC. Completeness and contamination percentages were determined using CheckM via BC-BRC (7). Biosynthetic gene clusters (BGCs) were predicted using antiSMASH 4.0 (8). Genome summary statistics are described in Table 1.

**TABLE 1:** Summary genome statistics

Isolate accession	Patient characteristics	Gastric biopsy characteristics	No. of contigs	N50 (bp)	Coverage (×)	Genome size (bp)	GC content (%)	Predicted no. of: ^*a*^^,*b*^			CheckM		GenBank accession no.	SRA accession no.
								Proteins	tRNAs	rRNAs	Completeness (%)	Contamination (%)		
*S. epidermidis* 14-1777-C6	Male from Túquerres, Colombia (1.0857° N, 77.6186° W)	Non-atrophic gastric; positive for *H. pylori*	62	103,594	85.9	2,482,584	31.9	2,377	58	9	100	0	NXMC00000000	SRR35278348
*S. salivarius* 14-1770-C1	Female from Tumaco, Colombia (1.8087° N, 78.7667° W)	Non-atrophic gastric; negative for *H. pylori*	45	149,736	59.6	2,355,730	39.3	2,245	49	6	99.8	2.1	NXMB00000000	SRR35278347
*S. anginosus* MIT 14-1787-A7	Male from Túquerres, Colombia (1.0857° N, 77.6186° W)	Multifocal atrophic gastritis; positive for *H. pylori*	31	146,829	185.3	1,846,644	38.8	1,859	29	3	100	0	JBQWSQ000000000	SRR35278346

^*a*^ Number of gene sequences predicted using RASTtk.

^*b*^ RASTtk annotations available at https://www.bv-brc.org/view/Genome/1282.2266 for *S. epidermidis *14-1777-C6, https://www.bv-brc.org/view/Genome/1304.267 for *S. salivarius* 14-1770-C1, and https://github.com/TonyMannion/Novel-Staphylococcus-and-Streptococcus-strains-from-human-gastric-microbiome for *S. anginosus* MIT 14-1787-A7.

Taxonomic classification of genomes was supported by average nucleotide identity (pyANI, version: 0.2.10) (9) and digital DNA-DNA hybridization (Genome-to-Genome Distance Calculator, version 3.0) (10) analyses against reference strains in BV-BRC. Phenol-soluble modulins (PSMα, PSMβ1a, PSMβ1b, PSMβ2, PSMβ3, PSMδ, and PSMε) and δ-toxin (*hld*) with its associated accessory gene regulator quorum-sensing system proteins (*argBDCA*) were identified in *S. epidermidis*. These products are cytotoxic peptides that facilitate pathogenic infection through immune activation (11, 12), antimicrobial activity (13–15), and promotion of biofilm formation (16). Three different lantibiotic bacteriocin BGCs were identified in *S. salivarius* that had synteny with salivaricin 9, salivaricin M, and salivaricin A2 found in the probiotic strain *S. salivarius* M18. Salivaricin products have been reported to have antibacterial effect against *Lactococcus lactis*, *Enterococcus hirae*, *Staphylococcus aureus*, and other *Streptococcus* species (16, 17). These antibacterial peptides may modulate microbiome composition and function. The genome of *S. anginosus* harbors TMPC, a surface protein virulence factor that interacts with Annexin A2 receptor on gastric epithelial cells to promote bacterial attachment and colonization as well as induce mitogen-activated protein kinase activation which may contribute to gastric carcinogenesis (5).

In conclusion, *S. epidermidis*, *S. salivarius*, and *S. anginosus* strains represent novel strains from the gastric microbiota with genetic factors that may influence host susceptibility for gastric disease and cancer. Additional studies are needed to fully characterize the role of the strains in disease etiology and pathogenesis.

## Data Availability

Genomes and sequencing reads have been deposited in GenBank and SRA database under the accession numbers described in Table 1. Genomes submitted to GenBank were automatically annotated by PGAP.

## References

[1] Mannion A, Sheh A, Shen Z, Dzink-Fox J, Piazuelo MB, Wilson KT, Peek R, Fox JG. 2023. Shotgun metagenomics of gastric biopsies reveals compositional and functional microbiome shifts in high- and low-gastric-cancer-risk populations from Colombia, South America. Gut Microbes 15:2186677. doi:10.1080/19490976.2023.2186677 36907988 PMC10026914

[2] Shen Z, Dzink-Fox J, Feng Y, Muthupalani S, Mannion AJ, Sheh A, Whary MT, Holcombe HR, Piazuelo BM, Bravo LE, Josenhans C, Suerbaum S, Wilson KT, Peek RM, Wang TC, Fox JG. 2022. Gastric non-helicobacter pylori urease-positive Staphylococcus epidermidis and Streptococcus salivarius Isolated from humans have contrasting effects on H. pylori-associated gastric pathology and host immune responses in a murine model of gastric cancer. mSphere 7:e00772–21. doi:10.1128/msphere.00772-21 35138124 PMC8826947

[3] Yang I, Woltemate S, Piazuelo MB, Bravo LE, Yepez MC, Romero-Gallo J, Delgado AG, Wilson KT, Peek RM, Correa P, Josenhans C, Fox JG, Suerbaum S. 2016. Different gastric microbiota compositions in two human populations with high and low gastric cancer risk in Colombia. Sci Rep 6:18594. doi:10.1038/srep18594 26729566 PMC4700446

[4] Shen Z, Dzink-Fox J, Boucher M, Feng Y, Ge Z, Mannion AJ, Piazuelo BM, Peek RM, Wilson KT, Fox JG. 2025. Streptococcus anginosus induces gastric inflammation in germ free INS-GAS mice. 38th Int. Workshop Eur. Helicobacter Microbiota Study Group EHMSG 2025.

[5] Fu K, Cheung AHK, Wong CC, Liu W, Zhou Y, Wang F, Huang P, Yuan K, Coker OO, Pan Y, Chen D, Lam NM, Gao M, Zhang X, Huang H, To KF, Sung JJY, Yu J. 2024. Streptococcus anginosus promotes gastric inflammation, atrophy, and tumorigenesis in mice. Cell 187:882–896. doi:10.1016/j.cell.2024.01.004 38295787

[6] Bushnell B. 2014. BBMap: a fast, accurate, splice-aware aligner. 9th Annual Genomics of Energy & Environment Meeting BBTools Software Package. https://bbmap.org.

[7] Olson RD, Assaf R, Brettin T, Conrad N, Cucinell C, Davis JJ, Dempsey DM, Dickerman A, Dietrich EM, Kenyon RW, et al.. 2023. Introducing the Bacterial and Viral Bioinformatics Resource Center (BV-BRC): a resource combining PATRIC, IRD and ViPR. Nucleic Acids Res 51:D678–D689. doi:10.1093/nar/gkac1003 36350631 PMC9825582

[8] Blin K, Wolf T, Chevrette MG, Lu X, Schwalen CJ, Kautsar SA, Suarez Duran HG, de Los Santos ELC, Kim HU, Nave M, Dickschat JS, Mitchell DA, Shelest E, Breitling R, Takano E, Lee SY, Weber T, Medema MH. 2017. antiSMASH 4.0-improvements in chemistry prediction and gene cluster boundary identification. Nucleic Acids Res 45:W36–W41. doi:10.1093/nar/gkx319 28460038 PMC5570095

[9] Pritchard L, Glover RH, Humphris S, Elphinstone JG, Toth IK. 2016. Genomics and taxonomy in diagnostics for food security: soft-rotting enterobacterial plant pathogens. Anal Methods 8:12–24. doi:10.1039/C5AY02550H

[10] Meier-Kolthoff JP, Auch AF, Klenk H-P, Göker M. 2013. Genome sequence-based species delimitation with confidence intervals and improved distance functions. BMC Bioinformatics 14:60. doi:10.1186/1471-2105-14-60 23432962 PMC3665452

[11] Kretschmer D, Gleske A-K, Rautenberg M, Wang R, Köberle M, Bohn E, Schöneberg T, Rabiet M-J, Boulay F, Klebanoff SJ, van Kessel KA, van Strijp JA, Otto M, Peschel A. 2010. Human formyl peptide receptor 2 senses highly pathogenic Staphylococcus aureus. Cell Host Microbe 7:463–473. doi:10.1016/j.chom.2010.05.012 20542250 PMC3417054

[12] Nakamura Y, Oscherwitz J, Cease KB, Chan SM, Muñoz-Planillo R, Hasegawa M, Villaruz AE, Cheung GYC, McGavin MJ, Travers JB, Otto M, Inohara N, Núñez G. 2013. Staphylococcus δ-toxin induces allergic skin disease by activating mast cells. Nature 503:397–401. doi:10.1038/nature12655 24172897 PMC4090780

[13] Cogen AL, Yamasaki K, Sanchez KM, Dorschner RA, Lai Y, MacLeod DT, Torpey JW, Otto M, Nizet V, Kim JE, Gallo RL. 2010. Selective antimicrobial action is provided by phenol-soluble modulins derived from Staphylococcus epidermidis, a normal resident of the skin. J Invest Dermatol 130:192–200. doi:10.1038/jid.2009.243 19710683 PMC2796468

[14] Joo H-S, Cheung GYC, Otto M. 2011. Antimicrobial activity of community-associated methicillin-resistant Staphylococcus aureus is caused by phenol-soluble modulin derivatives. J Biol Chem 286:8933–8940. doi:10.1074/jbc.M111.221382 21278255 PMC3059065

[15] Cogen AL, Yamasaki K, Muto J, Sanchez KM, Crotty Alexander L, Tanios J, Lai Y, Kim JE, Nizet V, Gallo RL. 2010. Staphylococcus epidermidis antimicrobial delta-toxin (phenol-soluble modulin-gamma) cooperates with host antimicrobial peptides to kill group A Streptococcus. PLoS One 5:e8557. doi:10.1371/journal.pone.0008557 20052280 PMC2796718

[16] Periasamy S, Joo H-S, Duong AC, Bach T-HL, Tan VY, Chatterjee SS, Cheung GYC, Otto M. 2012. How Staphylococcus aureus biofilms develop their characteristic structure. Proc Natl Acad Sci USA 109:1281–1286. doi:10.1073/pnas.1115006109 22232686 PMC3268330

[17] Barbour A, Wescombe P, Smith L. 2020. Evolution of lantibiotic salivaricins: new weapons to fight infectious diseases. Trends Microbiol 28:578–593. doi:10.1016/j.tim.2020.03.001 32544444

